# Hierarchical Genetic Analysis of German Cockroach (*Blattella germanica*) Populations from within Buildings to across Continents

**DOI:** 10.1371/journal.pone.0102321

**Published:** 2014-07-14

**Authors:** Edward L. Vargo, Jonathan R. Crissman, Warren Booth, Richard G. Santangelo, Dmitry V. Mukha, Coby Schal

**Affiliations:** 1 Department of Entomology and W. M. Center for Behavioral Biology, North Carolina State University, Raleigh, North Carolina, United States of America; 2 Vavilov Institute of General Genetics, Russian Academy of Sciences, Moscow, Russia; Virginia Tech, United States of America

## Abstract

Understanding the population structure of species that disperse primarily by human transport is essential to predicting and controlling human-mediated spread of invasive species. The German cockroach (*Blattella germanica*) is a widespread urban invader that can actively disperse within buildings but is spread solely by human-mediated dispersal over longer distances; however, its population structure is poorly understood. Using microsatellite markers we investigated population structure at several spatial scales, from populations within single apartment buildings to populations from several cities across the U.S. and Eurasia. Both traditional measures of genetic differentiation and Bayesian clustering methods revealed increasing levels of genetic differentiation at greater geographic scales. Our results are consistent with active dispersal of cockroaches largely limited to movement within a building. Their low levels of genetic differentiation, yet limited active spread between buildings, suggests a greater likelihood of human-mediated dispersal at more local scales (within a city) than at larger spatial scales (within and between continents). About half the populations from across the U.S. clustered together with other U.S. populations, and isolation by distance was evident across the U.S. Levels of genetic differentiation among Eurasian cities were greater than those in the U.S. and greater than those between the U.S. and Eurasia, but no clear pattern of structure at the continent level was detected. MtDNA sequence variation was low and failed to reveal any geographical structure. The weak genetic structure detected here is likely due to a combination of historical admixture among populations and periodic population bottlenecks and founder events, but more extensive studies are needed to determine whether signatures of global movement may be present in this species.

## Introduction

Human-mediated dispersal has greatly contributed to the spread of invasive and non-native species, distributing them far beyond their native ranges. Although long distance dispersal events, such as human transport, are typically rare, they can be important to population dynamics and the broad scale shaping of population genetic structure in some species [1]. Thus population genetic studies of species that disperse primarily by human transport can provide information on the role of human activity in the spread of invasive species and may allow identification of source populations and routes of transport which can be targeted to reduce the possibility of future introductions [2], [3], [4]. Moreover, understanding patterns of dispersal of medically-important arthropod and vertebrate species can have implications for managing the spread of vector-borne pathogens.

Urbanized areas provide many examples of human-mediated invasive species because of the considerable amount of transit between cities, and because cities provide modified environments and substantial resources that tend to favor invasive and non-native species at the expense of natives [5]. In consequence, many of the same invasive species are found in a large number of cities across wide geographic ranges [6]. These broadly distributed urban species can offer unique opportunities for examining the role that human-mediated dispersal plays in shaping population structure over a large geographic scale.

Genetic markers have long been recognized as useful tools for the study of population genetic structure for the inference of past demographic events, including dispersal and gene flow [7]. Evaluation of genetic structure between populations can allow detection of patterns of dispersal between established populations as well as the sources of invasion into new areas [8]. However, genetic studies of invasive populations pose particular challenges. Due to the nature of the invasion process, the relative youth of many invasive populations, and ongoing long-range dispersal of many invasive species, especially those that are close human commensals, invasive populations are not in mutation-migration-drift equilibrium making standard estimates of gene flow from *F*-statistics [9] inappropriate. However, Bayesian multilocus methods enable estimation of population structure and recent migration without assumption of mutation-migration-drift equilibrium (e.g., [10], [11], [12], [13]). Microsatellite markers, which typically exhibit high levels of variability, are well suited for such methods [14], as well as providing powerful tests of population differentiation [15].

The German cockroach (*Blattella germanica* L.) is a ubiquitous inhabitant of human-built structures and is widely considered one of the most prominent of the structural pests and arguably one of the most successful invasive species on earth. It has been implicated in the spread of numerous human pathogens, including bacteria (e.g., [16], [17], [18], [19]), fungi [20], and protozoa [21], [22]. *Blattella germanica* also plays a major role in the production and dissemination of household allergens [23]. Cockroach allergens can have a severe impact on human health by increasing the incidence of asthma morbidity, especially among inner-city children [24].

Human-mediated dispersal has been integral to the spread of the German cockroach, which is now established in cosmopolitan regions on every continent [25]. It is also one of the most effective exploiters of urban environments. In fact, *B. germanica* is so specifically adapted to human-built structures that it has become an obligate commensal species and is not known to exist outdoors or in natural environments anywhere in its distribution [26]. As such, the outdoors environment represents an effective barrier to active dispersal for the German cockroach, and any movement beyond buildings must be human-mediated. Nevertheless, because of its close association with human-built structures, the German cockroach readily utilizes human transportation vectors (i.e., buses, trains, ships, airplanes) to disperse locally and globally.

Little evidence exists on the broad-scale population genetic structure of the German cockroach. Cloarec *et al*. [27] sampled from 31 *B. germanica* populations from two cities in France and analyzed them using allozymes. These authors found strong levels of genetic differentiation among populations within each city but no evidence for differentiation between cities even though they were located 900 km apart. A follow-up study [28] using two *B. germanica* populations from each of the same two French cities found significant genetic differentiation between them at RAPD loci. However, principal component analysis was unable to separate the four populations according to city. Although these results may be explained in part by the use of relatively low diversity allozyme markers and the unreliability of RAPD markers (e.g., [29], [30]) respectively, these two studies showed a surprising lack of genetic structure between cities relative to within cities, as would be expected if cockroaches were moved around more frequently within a city than between cities.

Recently, the role of potential transportation routes in structuring German cockroach populations in swine farms in southeastern North Carolina has been investigated. Previously, Mukha et al. [31] compared *B. germanica* populations from three North Carolina swine farms using restriction fragment length polymorphisms of the non-transcribed spacer region of ribosomal DNA. We observed greater genetic divergence between farms managed by different companies separated by only 15 km than between farms separated by >100 km but managed by the same company. This pattern was consistent with the hypothesis that gene flow would be facilitated by human-mediated movement within each company's supply chain. To test this hypothesis, Booth et al. [32] studied cockroach populations on 22 farms using microsatellite markers. Their results showed significant differentiation among farms, but this variation was more associated with geographic proximity, and therefore human-mediated dispersal on a local scale, rather than dispersal through movement by management companies.

On a finer geographic scale, we previously investigated the population genetic structure of German cockroach infestations within and among apartment buildings in Raleigh, North Carolina using microsatellite markers [33]. Within each apartment, individuals formed a single panmictic population with no genetic differentiation among aggregations from different rooms. However, cockroaches collected in different apartments in the same building were significantly differentiated, and apartments in different nearby buildings showed even stronger genetic differentiation.

Our goal here was to expand on previous *B. germanica* studies by sampling from a greater number of populations, and by comparing genetic structure across a range of spatial scales from populations in apartments within the same building to populations on different continents to better understand the potential range of human-mediated dispersal. We aimed to improve resolution relative to previous German cockroach studies in urban environments at the level of the city and above by employing high diversity species-specific microsatellite markers [34]. We expected genetic structure to be hierarchical, with the degree of genetic similarity being greater within a city than among cities due to common ancestry or recent gene flow, and greater within the U.S. than between continents.

## Results

### Genetic Diversity and Spatial Scale Comparisons

Levels of genetic diversity were highly variable among the 34 U.S. and Puerto Rico samples with observed heterozygosities ranging from 0.46 in the Hickory, NC (HNC) population to 0.76 in the LS-B Raleigh, NC population (Table 1). Observed heterozygosities were significantly lower than expected heterozygosities (*t*_33_ = −4.97, *P*<0.0001, paired *t*-test), suggesting populations were slightly inbred. Mean number of alleles per locus ranged from 4.62 in the Hickory, NC population to 8.50 in the Gainesville, FL (GFL) population and allelic richness (based on 8 diploid individuals) ranging from 3.36 in the Hickory, NC population to 5.74 in the Puerto Rico (PR) population. Genetic diversity was generally much lower in the *B. germanica* populations from Eastern Europe and Asia, with only Singapore (SIN) having a level of heterozygosity and average alleles per locus comparable to the U.S. populations. As with the U.S. populations, observed heterozygosities were significantly lower than expected (*t*_5_ = −3.10, *P*<0.03, paired *t*-test). In all other populations, observed heterozygosity and alleles per locus were as low as 0.42 and 3.25, respectively, despite sample sizes of 30 individuals in each case. While eight of 18 Raleigh populations deviated significantly from Hardy-Weinberg equilibrium, only three of the other 16 U.S. samples differed significantly from the expected frequencies. All Eurasian samples except the Kiev (KUA) and Crimea (CUA) populations, which had the lowest average number of alleles per locus in this group, tested highly significantly (*P*<0.001) for departure from Hardy-Weinberg equilibrium.

**Table 1:** Sampling information and diversity statistics across eight loci for *B. germanica* populations.

Location	Code	*n*	*N*_A_	*A*_R_	*H*_E_	*H*_O_	HWE^b^
*North America*							
Raleigh, NC^a^							
Apartments within a building^c^	LS-A	29	7.87	5.55	0.75	0.74	ns
	LS-B	30	8.00	5.44	0.76	0.76	ns
	LS-C	30	7.50	5.31	0.75	0.66	***
	CR-A	30	8.12	5.62	0.76	0.74	**
	CR-B	30	8.12	5.47	0.74	0.66	***
	CR-C	29	8.00	5.61	0.75	0.71	**
	CS-A	30	8.37	5.42	0.74	0.69	ns
	CS-B	29	8.12	5.61	0.74	0.75	ns
	CS-C	30	6.87	4.69	0.70	0.63	ns
Apartments in different buildings^c^	DR-X	30	7.75	5.26	0.70	0.63	ns
	DR-Y	30	7.87	5.24	0.72	0.68	***
	DR-Z	30	7.87	5.32	0.69	0.67	ns
	DRD-X	30	6.75	4.89	0.71	0.71	***
	DRD-Y	30	7.62	5.39	0.73	0.72	ns
	DRD-Z	30	6.75	4.78	0.69	0.72	ns
	FC-X	30	8.12	5.36	0.74	0.71	***
	FC-Y	30	6.87	5.07	0.69	0.65	ns
	FC-Z	30	7.50	5.08	0.68	0.61	*
Norfolk, VA	NVA	30	6.25	4.68	0.70	0.73	ns
Richmond, VA	RVA	30	6.25	4.56	0.67	0.66	ns
Hickory, NC	HNC	30	4.62	3.36	0.53	0.46	***
Griffin, GA	GGA	29	6.37	5.06	0.68	0.64	ns
Bryan, TX	BTX	30	7.12	5.18	0.71	0.67	ns
Baton Rouge, LA	BRL	22	7.25	5.25	0.68	0.56	*
White Eagle, OK	WOK	29	8.00	5.68	0.72	0.74	ns
Gary, IN	GIN	30	7.75	5.33	0.69	0.62	ns
Orange, CA	OCA	8	3.37	3.37	0.54	0.55	ns
Los Angeles, CA	LCA	13	5.87	5.06	0.71	0.67	ns
Compton, CA	CCA	29	7.37	5.07	0.72	0.63	ns
Cleveland, OH	COH	27	5.50	3.91	0.62	0.61	ns
Gainesville, FL	GFL	28	8.50	5.48	0.73	0.73	ns
Minneapolis, MN	MMN	30	7.87	5.25	0.71	0.55	***
Hawaii	HAW	10	3.87	3.71	0.62	0.58	ns
Puerto Rico	PR	30	8.12	5.74	0.74	0.73	ns
US and Puerto Rico Mean			7.13	5.05	0.70	0.66	
*Eurasia*							
Moscow, Russia	MRU	30	3.87	3.27	0.56	0.48	***
Tomsk, Russia	TRU	30	5.87	4.37	0.59	0.52	***
Kiev, Ukraine	KUA	30	3.25	2.65	0.51	0.50	ns
Crimea, Ukraine	CUA	30	3.62	3.07	0.51	0.52	ns
Tehran, Iran	TIR	30	4.12	3.25	0.49	0.42	***
Singapore	SIN	30	8.25	5.85	0.78	0.70	***
Eurasia mean			4.83	3.74	0.57	0.52	

Mean number of alleles per locus (*N*_A_), allelic richness (*A*_R_), expected heterozygosity (*H*_E_), observed heterozygosity (*H*_O_), and results for Hardy-Weinberg equilibrium tests (HWE).

a LS, CR, CS, DR, DRD and FC are six different apartment complexes; A, B, and C represent separate apartments within individual buildings; X, Y and Z represent separate apartments in different buildings in the same apartment complex.

b Significance of Hardy-Weinberg tests [15]: *P<0.05, **P<0.01, ***P<0.001; ns, not significant.

c Samples were the same as those used in Crissman et al [33].

Pairwise tests of differentiation showed that in all but 15 of the 780 comparisons, populations were significantly differentiated following Bonferroni correction (*P*<0.0001). In the 15 cases in which populations were not significantly differentiated, 12 involved populations in Raleigh; in five cases it involved apartments within the same building (LS-A, B, and C were not differentiated, nor was CS-A and CS-B or CS-B and CS-C) and in two cases it involved apartments in the same complex but different buildings (DR-X was not significantly differentiated from DR-Y or DR-Z). The remaining three cases involved population CCA (Compton, CA), which was not significantly differentiated from three populations in Raleigh: DR-X, DRD-Y and CS-A.

**Figure 1:**
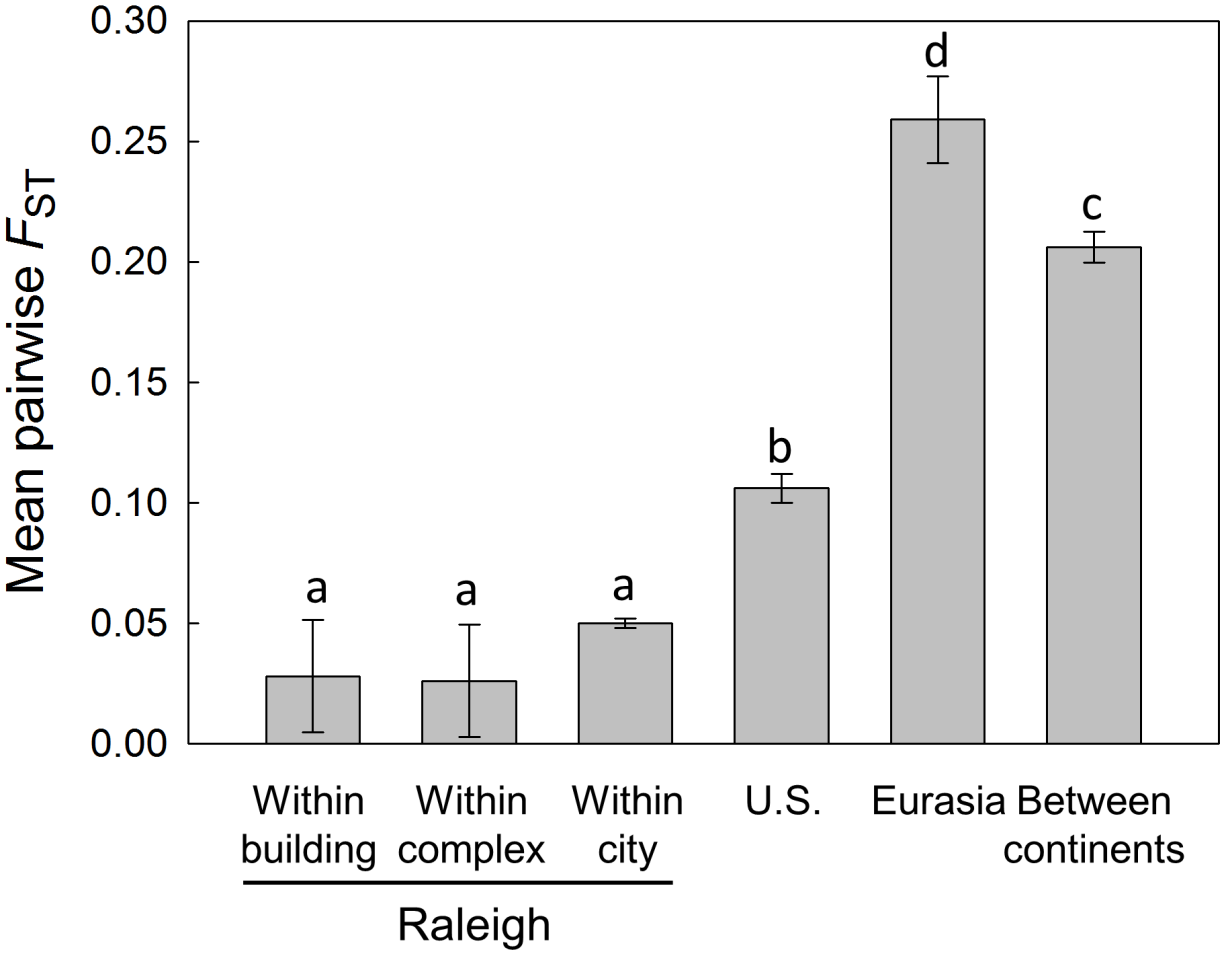
Levels of genetic differentiation among *B. germanica* populations at different spatial scales. Shown are mean pairwise *F*_ST_ values (±SE) for all pairs of populations at each level of analysis. The value for the U.S. consists of comparisons between cities in the continental U.S. (i.e., excluding Hawaii and Puerto Rico), including comparisons between Raleigh and other cities but excluding comparisons between apartments within Raleigh represented by only sample location DR-X. The value for “between continents” consists of all comparisons between populations in North America and populations in Eurasia. Bars labeled with different letters are significantly different (*P*<0.05).

An ANOVA test found significant differences (*F*_5,415_ = 112.36, *P*<0.0001) in mean pairwise *F*_ST_ values across our six spatial scales (Fig. 1), and a Tukey-Kramer test found that all means above the level of the city were significantly different from each other, whereas at scales of the city level and below, including levels of differentiation among apartments in the same building, there were no significant differences. Mean pairwise *F*_ST_ values (SE) increased from 0.028 (0.023) and 0.026 (0.023) within apartment buildings and apartment complexes, respectively to 0.050 (0.002) among locations within Raleigh, to 0.106 (0.006) across U.S. populations using only a single population from Raleigh (DR-X), and to 0.206 (0.006) when considering only comparisons between continents. However, mean *F*_ST_ was significantly higher within Eurasia, with a value of 0.259 (0.018). Within the U.S. (excluding comparisons among Raleigh populations), the least differentiated pair of populations (*F*_ST_ = 0.025) was Norfolk, VA (NVA) and Raleigh, NC (DR-Y), and Baton Rouge, LA (BTL) and Raleigh, NC (CS-A), whereas the most differentiated pair of populations (*F*_ST_ = 0.350) was Hickory, NC (HNC) and Gary, IN (GIN). Within Eurasia, the least differentiated pair of populations (*F*_ST_ = 0.181) was TRU (Tomsk, Russia) and TIR (Tehran, Iran), whereas the most differentiated pair of populations (*F*_ST_ = 0.335) was MRU (Moscow, Russia) and TIR (Tehran, Iran). Comparing the U.S. and Eurasia, the least differentiated pair of populations (*F*_ST_ = 0.053) was Raleigh, NC (DRD-Y) and Singapore (SIN), whereas the most differentiated pair (*F*_ST_ = 0.391) was Hickory, NC (HNC) and Crimea, Ukraine (CUA).

**Figure 2:**
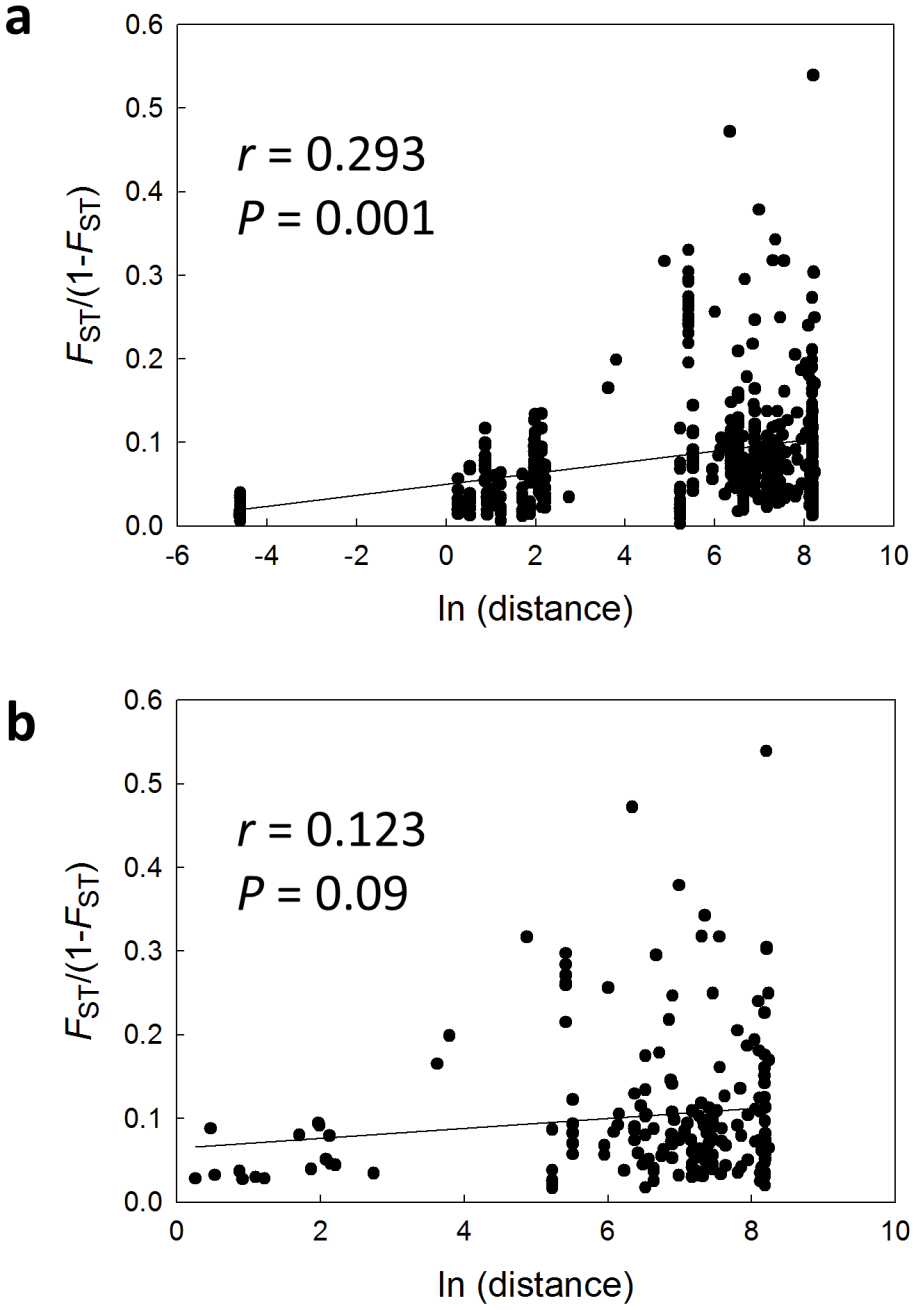
Isolation by distance analysis for *B. germanica* populations across the U.S. Pairwise comparisons between populations are plotted as genetic distance transformed as *F*_ST_/(1-*F*_ST_) versus the natural log of geographic distance. The correlation coefficient (*r*) and results for the Mantel test of significance are given for (a) all U.S. populations, including comparisons between apartments within the same complex in Raleigh, North Carolina; and (b) for comparisons between populations in the continental U.S. using six populations from Raleigh, one population from each of the studied apartment complexes (LS-A, CR-A, CS-A, DR-X, DRD-X, FC-X).

The Mantel test for all continental U.S. samples, used to determine whether genetic differentiation and geographic distance were correlated, was significant when all Raleigh samples were included to capture the full range of spatial scales (*P* > 0.001), although the slope of the regression was only 0.007 (Fig. 2a). When Raleigh was represented by only six populations, one from each apartment complex, the Mantel test showed no significant isolation by distance for comparisons between populations within the continental U.S. cities (*P* = 0.09; Fig. 2b).

### Bayesian Clustering and Admixture Analysis

**Figure 3:**
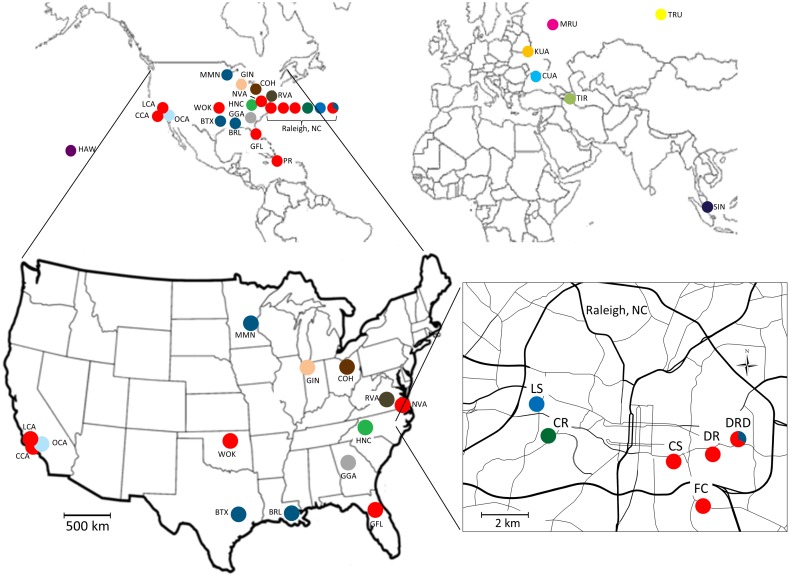
Bayesian cluster analysis of 40 global *B. germanica* populations as identified by the program BAPS. Populations given the same color were grouped into the same genetic cluster. The analysis identified 17 distinct clusters, 11 for the 36 U.S. populations, whereas each of the six Eurasian populations formed a unique cluster. The 18 Raleigh, North Carolina samples are grouped by apartment complex. In only one case (complex DRD) was an apartment not clustered together with populations from apartments in the same apartment complex. A finer resolution map showing the location of the Raleigh, NC populations is given in Crissman et al. [33].

The BAPS analysis returned 17 partitions (clusters) as shown in Fig. 3 and Table 2. Of the 17 identified clusters, 13 consisted of a single sample location. All of the clusters containing multiple populations were comprised of U.S. populations only, with only five of these populations showing the presence of some significantly admixed individuals, including two populations from the CR apartment complex in Raleigh (Table 2). Cluster 2 was the largest cluster, with 17 populations, 11 of which came from Raleigh, NC. The Raleigh populations were partitioned into four clusters (Clusters 1–4). Each set of apartments from the same building was placed into the same genetic cluster, but each set formed a different cluster. Samples LS and CR formed unique clusters, whereas sample CS was part of a large genetic cluster that included nearly all the remaining Raleigh samples as well as samples from Norfolk, VA (NVA), White Eagle, OK (WOK), Los Angeles, CA (LCA), Compton, CA (CCA), Gainesville, FL (GFL) and Puerto Rico (PR). All of the apartment samples collected from separate buildings in the same complex (samples DR, DRD and FC) were clustered together with the exception of sample DRD-Z which grouped together with samples from Bryan, TX (BTX), Baton Rouge, LA (BRL) and Minneapolis, MN (MMN). For the Eurasian samples, each population formed a unique cluster, with only the Kiev, Ukraine (KUA) and Singapore (SIN) populations showing significant admixture.

**Table 2:** Results from the Bayesian admixture analysis showing the grouping of populations into clusters and the proportion of significantly admixed individuals (*P*<0.05) in each population.

Cluster	Population	Admixture
1	DRD-Z	0
	BTX	0
	BRL	0
	MMN	0
2	DR-X	0
	DR-Y	0
	DR-Z	0
	DRD-X	0
	DRD-Y	0
	FC-X	0
	FC-Y	0
	FC-Z	0
	CS-A	0
	CS-B	0
	CS-C	0
	NVA	0
	WOK	0
	LCA	0
	CCA	0
	GFL	0
	PR	0
3	LS-A	0
	LS-B	0
	LS-C	0
4	CR-A	0
	CR-B	0.067
	CR-C	0.300
5	RVA	0
6	HNC	0.033
7	GGA	0
8	OCA	0
9	COH	0.037
10	GIN	0.067
11	HAW	0
12	MRU	0
13	TRU	0
14	KUA	0.033
15	CUA	0
16	TIR	0
17	SIN	0.133

### Mitochondrial 16S

A total of 66 individuals from 34 populations were sequenced for the 16S mtDNA gene. The sequences have been deposited in GenBank (accession numbers KJ937895-KJ937960). Only three sites were variable within the 385 bp region examined (0.8% uncorrected sequence variation), and the average nucleotide difference was only 2.63. A parsimony haplotype network computed in *TCS* found a maximum of eight connection steps within a probability limit of 95% (*P* = 0.954), with one haplotype not connecting to the others within this limit (not shown). Haplotypes varied greatly in their frequency and geographic ranges, and no geographic pattern of *B. germanica* mitochondrial diversity was detectable with this network. Given the low degree of sequence variation, no further individuals were sequenced for this gene.

## Discussion

Using a combination of standard measures of genetic differentiation and Bayesian clustering methods, we characterized the genetic structure of German cockroach populations at various spatial scales from within buildings to across continents. Our results show increasing levels of genetic differentiation among apartment infestations at greater spatial scales, but also reveal a complex structure that reflects human-mediated dispersal across both short and long distances. Genetic differentiation as measured by *F*_ST_ was lowest among samples collected from the same apartment building and among populations from different buildings in the same housing complex, although the average values were not significantly different from those among populations at the within-city level. Results of the Bayesian analysis revealed a greater level of structure within 10 km within the city of Raleigh than was evident from *F*-statistics alone, placing each set of apartments from the same building into the same genetic clusters, but each set formed a different cluster. In addition, all of the apartment samples collected from separate buildings in the same complex (samples DR, DRD and FC) were clustered together except for sample DRD-Z which grouped together with samples from Texas and Minnesota.

Taken as a whole, our results show little genetic differentiation among samples collected from different apartments in the same building, whereas there was evidence of slightly greater levels of differentiation among populations from different buildings in the same apartment complex. These results are consistent with the limited active dispersal range of this flightless cockroach [33], [35], [36], and suggest that while active dispersal of cockroaches within a building may be extensive, movement among buildings, even those located near each other, is much more restricted.

At the within-city scale, we found a mosaic pattern of genetic differentiation. Bayesian analysis detected four genetic clusters in the city of Raleigh, with nearly all of the populations from four of the six apartment complexes clustered together with populations from more distant locations, including California and Puerto Rico. This heterogeneous mix of apparently similar and dissimilar populations within a city is consistent with limited active dispersal between buildings and extensive human-mediated dispersal through movement of infested materials within the city as well as between more distant locations. While we found some clustering of populations within the city of Raleigh, previous genetic studies of *B. germanica* using allozymes and RAPDs did not find evidence of population clustering in two French cities located approximately 900 km apart [27], [28].

Levels of pairwise differentiation among the six Eurasian populations were higher than those among U.S. populations. This may be due in part to the fact that the Eurasian samples represented a larger geographic area than the samples from the U.S., although in the two cases where there were samples from the same country within Eurasia, pairwise *F*_ST_-values were relatively high (Moscow and Tomsk, Russia, *F*_ST_ = 0.261, distance 2900 km; and Kiev and Crimea, Ukraine, *F*_ST_ = 0.315, distance 673 km). Nonetheless, the degree of genetic differentiation within Eurasia was greater than that between the U.S. and Eurasia.

We were able to detect very little population genetic structure differentiating German cockroach populations as a whole between the different continents. We did find that mean *F*_ST_ values within the U.S. were significantly lower than those between continents (Fig. 1), and while some U.S. populations clustered together in the BAPS analysis, the Eurasian populations each formed distinct clusters. The results suggest greater movement of cockroaches among cities in the U.S. or a more recent common ancestry than in the Eurasian populations, although the sample size for the latter area was small (*n* = 6) and represented a very large geographic region and several different countries. In addition, we found little divergence in global samples for the mitochondrial 16S gene, and no geographical pattern of haplotype diversity. These results suggest that there is some historical connectivity between populations around the world, a finding consistent with the centuries-long global movement of this species [37].

One possible explanation for the weakness of the global genetic structure is that a human-adapted lineage of German cockroaches was rapidly spread by human activity from a single source population with limited haplotype diversity, resulting in a relative lack of diversity on a global scale. This would be similar to the proposed expansion of a low-genetic diversity “ship rat” lineage of the black rat (*Rattus rattus*) across the globe [38]. The cockroach and the rat likely have a similar history of colonization, with introductions to North America around the time of Columbus's voyages. Much more recent colonizations can result in a similar genetic signature, as for example the commensal brown widow spider, which shows a surprisingly low level of global diversity relative to several congeners, possibly due to a recent human-mediated range expansion [39]. Another possibility is that there may have been a number of native populations that served as the sources for the global spread of *B. germanica*, but mtDNA diversity in the original source populations was low to begin with and human transport of cockroaches has homogenized global populations. We found only 0.8% sequence variation in the 16S gene across 34 populations. Similarly low mtDNA variation (0.5–0.8% nucleotide diversity) has been found in both native and introduced ranges of the invasive Formosan subterranean termite *Coptotermes formosanus* [40], [41], despite relatively high genetic diversity at nuclear microsatellite loci [42]. Distinguishing between these two possibilities will require the genetic characterization of one or more native populations of *B. germanica*, which are likely to be located in eastern Asia [26].

Genetic diversity among the studied *B. germanica* populations was relatively high, especially in the U.S., with a mean of 7.1 alleles per locus. Genetic diversity was somewhat lower in the Eurasian populations; the mean number of alleles and allelic richness were 32.2% and 26.0% lower than the U.S. populations, respectively. Expected and observed heterozygosity were also about 20% lower in Eurasia. Genetic diversity in the U.S. study populations, all of which came from human dwellings, was greater than that reported for 22 swine farms in southeastern North Carolina (mean no. alleles 5.47; *H*_E_ = 0.657; *H*_O_ = 0.626) based on the same eight microsatellite loci used here and with similar sample sizes (mean  = 28.2 individuals per population [32]). The lower genetic diversity in swine farm populations exists despite the fact that *B. germanica* populations in these farms can reach tens of thousands of individuals [43]. These results suggest that propagule sizes are larger in urban infestations than in agricultural structures, and/or that propagule pressure is greater in urban areas resulting in more frequent introductions into human dwellings.

Unlike our study, Booth et al. [32] did not find significant isolation by distance among German cockroach populations among swine farms in southeastern North Carolina. Similarly, Cloarec *et al*. [27] did not find significant isolation by distance when comparing German cockroach populations in two French cities. However, our study involved samples from across the U.S. and thus encompassed a much larger geographic area than either of the two previous studies. Similar to our results, weak but significant isolation by distance has been detected in the common bed bug, *Cimex lectularius*, another globally-distributed human commensal spread through human-mediated dispersal. Also using microsatellite markers, Saenz et al. [44] found a positive correlation (*r* = 0.072; *P*<0.0001) between pairwise *F*_ST_-values and geographic distance among 21 bed bug populations spanning the eastern U.S. from Florida to Massachusetts. Significant isolation by distance has also been reported in the black rat (*R. rattus*) in the U.S., which was likely introduced to the U.S. about the same time as *B. germanica* (mid 1500 s), but not in the Norway rat (*R. norvegicus*), which was introduced to the U.S. in the 1700 s [45].

Our results show similarities and differences to both the small-scale and large-scale genetic structure of *C. lectularius*, a species that has undergone very recent global resurgence. Using microsatellite markers, Booth et al. [46] found evidence of extensive spread of bed bug populations throughout infested apartment buildings. In the apartments studied by these authors, buildings appeared to be colonized by a small number of individuals, possibly a single female and/or her offspring in some cases. Thus, German cockroaches and bed bugs appear to spread to other apartments once they become established in multi-unit apartment buildings. However, genetic diversity in bed bug populations is much lower within infestations due to the small propagule size compared to the large numbers of German cockroaches introduced. Moreover, in two of the three apartment buildings studied by Booth et al. [46], there was evidence of significant genetic substructuring among bed bug populations in different apartments. In the two cases showing substructure, both in Jersey City, New Jersey, populations from different apartments clustered together into at least two distinct genetic groups, each group presumably arising from a separate propagule that expanded and then spread to multiple apartments with little or no admixture between the different clusters. These findings contrast with the present results on German cockroaches, although we only sampled three apartments per building, whereas the bed bug study sampled 5–17 apartments per building, so it is possible that our sample size was too small to detect possible genetic substructuring among *B. germanica* populations within a building. Alternatively, unique features of their respective mating systems might facilitate admixture and homogenization among cockroach propagules and not among bed bugs.

Genetic differentiation among populations in different buildings within the same apartment complex appears to be greater in bed bugs as well. Booth et al. [46] reported an *F*_ST_ of 0.179 between populations of *C. lectularius* in two apartment buildings in the same complex, whereas we found an average *F*_ST_ of only 0.026 among *B. germanica* populations located in different buildings in the same apartment complex. At spatial scales at the level of the city and above, bed bug populations show much greater differentiation than do German cockroach populations, with average pairwise *F*_ST_-values among populations in the eastern U.S. of 0.68 [44] compared to the average *F*_ST_ = 0.11 among *B. germanica* populations across of the U.S we found here. And, as mentioned above, weak but significant isolation by distance across large spatial scales has been found in both species. It is likely that the bed bug resurgence in the U.S. over the last 15–20 years has originated from multiple highly diverse source populations, with extensive propagule pressure and influx of new genotypes still occurring today. In contrast, German cockroaches have associated with the human-built environment in North America without interruption for >500 years. Global trade and transport likely have increased the frequency of long-distance dispersal events, facilitated gene flow, and homogenized populations, resulting in little global genetic structure in the German cockroach.

## Conclusions

We were able to detect very little global genetic structure in the German cockroach, which is probably attributable to a combination of many centuries of human transport and a lack of migration-drift equilibrium. However, traditional *F*-statistics, Bayesian clustering methods and isolation by distance did find a signal of greater connectivity of populations within a single city (Raleigh, NC) compared to connections among other cities in the U.S., and of populations within the U.S. relative to populations between continents, suggesting a greater history of gene flow and migration within each successively smaller spatial scale. On the other hand, cluster analyses provided evidence that long distance human-mediated dispersal of *B. germanica* frequently occurs in the U.S. resulting in a patchwork of local populations sharing recent ancestry intermixed with unrelated populations. We found evidence that there is greater movement of cockroaches among cities in the U.S. than in Eurasia but no clear pattern of global genetic structure from the regions we sampled. Mitochondrial DNA variation was very limited and therefore uninformative in revealing population structure. More extensive studies including a broader geographic range, particularly eastern Asia which is the likely geographic origin of *B. germanica*, should provide greater insights into the global genetic structure and dispersal history of this species.

## Materials and Methods

### Cockroach Collection

In order to address gene flow and differentiation at several spatial scales, cockroach samples were obtained for various geographic scales: among apartments within apartment buildings, among buildings within apartment complexes, among locations within a city, among cities across the U.S., and global (Table 1). Cockroaches were collected by pest control companies, collaborators, or by us and in all cases the resident or owner of the property gave permission to collect cockroaches from the site. Samples within a city were collected from three apartments in each of six low-income apartment complexes within Raleigh, North Carolina, from October to November 2006, May 2007, and December 2007 to January 2008 as described by Crissman et al. [33]. Genetic analysis of these samples was previously reported by Crissman et al. [33], and we follow the same sample designations here. Three apartments within a single low-rise building were sampled in each of the complexes LS, CR and CS. Two apartments, designated A and B, were adjoining and shared a wall, whereas the third apartment, labeled C, was located in another part of the same building. In complexes DR, DRD, and FC, three apartments were sampled from separate buildings, and these were designated as X, Y and Z (corresponding to A, B and C in Crissman et al. [33]).

An additional 24 samples of *B. germanica* for U.S. and global analysis were provided by colleagues; these samples represented 16 additional cities within the continental United States, Hawaii, Puerto Rico, and six international cities. Each sample represented a single collection from a single apartment at a single point in time. All samples were stored at −20°C in 95% ethanol until DNA extraction.

### DNA Extraction and Genotyping

DNA was extracted from the legs and thorax of each German cockroach using the Gentra Puregene DNA extraction kit (Qiagen, Germantown, MD). Each cockroach was amplified for 10 microsatellite loci (*Bg*-1D5, *Bg*-A7, *Bg*-B12, *Bg*-CO4, *Bg*-D05, *Bg*-D9, *Bg*-F7, *Bg*-G7, *Bg*-wb-1 A, *Bg*-wb-2 A) according to the polymerase chain reaction (PCR) procedures outlined by Booth et al. [34]. Loci *Bg*-wb-1 A and *Bg*-D9 were later removed from the analysis due to a high frequency of null alleles as detected by the program MICRO-CHECKER [47]. Amplified products were labeled with M13 IRDye infrared dyes (LI-COR Biosciences, Lincoln, NE) and run on 6.5% polyacrylamide gels using a LI-COR 4300 sequencer. Allele sizes were scored using Gene Profiler software version 4.05 (Scanalytics, Inc., BD Biosciences Bioimaging, Rockville, MD).

### Genetic Diversity and Summary Statistics

Mean number of alleles per locus, allelic richness (number of alleles per locus corrected for sample size), and expected and observed heterozygosity were calculated using FSTAT version 2.9.3.2 [48]. Departures from Hardy-Weinberg equilibrium and genotypic linkage equilibrium were tested for each sample across all loci in GENEPOP version 4.0 [15], [49] with a Bonferroni correction applied.

To determine if populations were significantly differentiated from each other, we conducted tests of genotypic differentiation for all pairs of populations using the exact χ^2^ test as implemented in GENEPOP [15], [49] with the Bonferroni correction applied. At each spatial scale, differentiation was assessed by global and pairwise *F*_ST_ values [50] computed using FSTAT. For estimating *F*_ST_ values across U.S. populations we used only a single population from Raleigh (DR-X). Confidence intervals for global *F*_ST_ values were provided by bootstrapping over loci. Pairwise *F*_ST_ values were compiled for all comparisons within each spatial scale and variance between scales was tested by ANOVA using JMP Pro 9.0.0 (SAS Institute Inc., Cary, NC). Mean *F*_ST_ values for each scale were compared using a Tukey-Kramer test.

Using a Mantel test implemented in GENEPOP, a test of isolation by distance was conducted to determine whether genetic differentiation and geographic distance were correlated across the U.S. The test was first conducted using all populations from within the continental U.S., including all 18 populations from within Raleigh (3 populations from each of 6 apartment complexes). Distances between apartment complexes in Raleigh ranged from 1.3–9.1 km, from 15–45 km for the three Los Angeles area populations, and from 130–3600 km for comparisons between cities. The Mantel test was performed a second time with comparisons of all populations using only a single population from each Raleigh apartment complex (*n* = 6; LS-A, CR-A, CS-A, DR-X, DRD-X, FC-X). Geographic distances were log_e_ transformed and genetic distances were transformed to *F*_ST_/(1-*F*_ST_), following Rousset [51].

### Bayesian Clustering

Partial Bayesian cluster analysis of predefined groupings (populations) was performed using BAPS 6.0 [10], [52] with the aim of identifying the optimum number of genetic clusters among groups of samples and the level of admixture among the different clusters. We used the Clustering of Individuals option with an upper bound of 40 populations. We followed the approach of Herborg et al. [53] to identify the proportion of individuals within a cluster showing significant evidence of admixture using the ‘nonequilbrium’ method of individual-based admixture as implemented in BAPS. We used the default values of 5 for the minimum size of a population, 50 iterations, 50 for the number of reference individuals per population, and 10 iterations per reference individual. The critical value of α for admixture was set at a posterior probability of 0.05.

### Sequencing of the Mitochondrial 16S Gene

Two randomly selected individuals per population were selected for preliminary mtDNA analysis and a 385 bp of the mtDNA 16S gene was sequenced. PCR was conducted using primers LR-J-13017 and LR-N-13398 [54] following the protocol of Xiong and Kocher [55]. We used two individuals per population based on previous results from sequencing 10 individuals per population that failed to yield more than a single haplotype (W. Booth, unpubl. data). Resulting products were purified using QIAquick PCR purification kit (Qiagen), labeled for sequencing reactions using BigDye Terminator cycle sequencing kit (Life Technologies, Carlsbad, CA) and sequenced using an Applied Biosystems 3730xl. Sequences were edited and forward and reverse sequences for each individual were assembled using the ContigExpress utility within Vector NTI Advance 10.3.0 (Life Technologies), and contigs were aligned using the AlignX component of Vector NTI Advance.

Aligned sequences for all global German cockroach populations were analyzed using *DnaSP* version 4.50.3 [56] to calculate the number of haplotypes and average number of nucleotide differences between sequences [57]. Haplotype networks for all populations were created using TCS [58] with the maximum number of allowable steps between haplotypes determined by a minimum parsimony probability of 95%.
